# From Evidence to Clinical Guidelines in Antibiotic Treatment in Acute Otitis Media in Children

**DOI:** 10.3390/antibiotics10010052

**Published:** 2021-01-06

**Authors:** Elena Lia Spoială, Gabriela Dumitrita Stanciu, Veronica Bild, Daniela Carmen Ababei, Cristina Gavrilovici

**Affiliations:** 1 Pediatrics Department, Grigore T. Popa University of Medicine and Pharmacy, 16 Universitatii Street, 700115 Iasi, Romania; lia_spoiala@yahoo.com (E.L.S.); cri.gavrilovici@umfiasi.ro (C.G.); 2 Center for Advanced Research and Development in Experimental Medicine (CEMEX), Grigore T. Popa University of Medicine and Pharmacy, 16 Universitatii Street, 700115 Iasi, Romania; veronica.bild@gmail.com; 3 Pharmacodynamics and Clinical Pharmacy Department, Grigore T. Popa University of Medicine and Pharmacy, 16 Universitatii Street, 700115 Iasi, Romania

**Keywords:** acute otitis media, clinical practice guidelines, antibiotic treatment, rodent models of acute otitis media, preclinical evaluation of antibiotics

## Abstract

Acute otitis media (AOM) in children represents a public health concern, being one of the leading causes of health care visits and antibiotic prescriptions worldwide. The overall aim of this paper is to unravel the major current insights into the antibiotic treatment of AOM in children. Our approach is three-fold: 1. a preclinical evaluation of antibiotics in animal models of AOM stressing on the advantages of different species when testing for different schemes of antibiotics; 2. an overview on the new antimicrobial agents whose efficacy has been demonstrated in refractory cases of AOM in children; and 3. an analysis of the different guidelines stressing on the differences and similarities between the various schemes of antibiotic treatment. The preferred therapeutic agents remain amoxicillin and the amoxicillin-clavulanate combination for AOM caused by *Streptococcus pneumoniae*, whereas oral cephalosporin is preferred in AOM due to *Moraxella catarrhalis* and *Haemophilus influenzae*. As for the second and third line antimicrobial treatments, there is a wide variety of suggested antibiotic classes with variations in duration and posology. The decision to prescribe antimicrobial treatment as a first-line choice is based on the severity of the symptoms in 16 of the guidelines included in this review.

## 1. Background

Acute otitis media (AOM) is a multifactorial disease representing the second most common cause of family physician visits in the pediatric population, following upper respiratory infections [1,2], with a negative economic impact [3]. Although we face nowadays a decrease in the number of otitis cases due to pneumococcal vaccination in infants [4], by 1 year of age about 23% of the children experience ≥1 episode of otitis media (OM), and by 3 years of age 60% experience ≥1 episode of OM and 24% ≥3 episodes [5]. Although the susceptibility to antibiotics among bacteria commonly causing AOM has decreased, the importance of antibiotic therapy cannot be denied [6,7]. In the pre-antibiotic era, 6% of the patients with AOM developed infectious complications [8], although the incidence of suppurated complications has declined to less than 1% after the introduction of antibiotics [9].

Our aim is to unravel the major insights into the antibiotic treatment of AOM in children. We will start by presenting a preclinical evaluation of antibiotics in animal models of AOM stressing on the advantages (characteristics) of different species when testing for different schemes of antibiotics. In the second part of our paper, we will provide an overview on the new antimicrobial agents whose efficacy has been demonstrated in refractory cases of AOM in children. Therapeutic failures in AOM in children are usually due to inadequate antimicrobial treatment, non-identification of the pathogen, or antimicrobial resistance [10,11,12]. To discuss each of these issues is beyond the purpose of our paper. We will refer only to antibiotic resistance because this is nowadays a frequent phenomenon. In the last part of the paper, we will analyze the different guidelines for the treatment of AOM in children, stressing on the differences and similarities between the various schemes of antibiotic treatment.

## 2. Preclinical Evaluation of Antibiotics in Animal Models of Acute Otitis Media

Animal models have been and will continue to be a powerful way to explore the mechanisms of diseases, with a significant impact on the development of new compounds and optimal therapeutic regimens [13]. Preclinical models of AOM in rodents allow the study of histopathological alterations during inflammation of the middle ear [14,15,16]; potential new therapies, including antibiotics or vaccines and the biocompatibility of implanted biomaterials [17,18]. Each AOM animal model (mice, rats, gerbils, chinchillas, or guinea pigs) studied over time has highlighted a series of drawbacks, but if the proposed limitations or questions are those to which the model can answer, the data acquired could be consistently interpreted [19]. Animal models of AOM revealed several histological and pathological similarities with the human disease, leading to the possibility of extrapolating the findings to new genetic or transgenic models. Moreover, the small size of most of these species facilitates low husbandry costs and easy handling (Table 1). Additionally, in vivo preclinical models utilized in laboratory settings frequently possess predictable pharmacokinetics, pharmacodynamics, and physiology, which have been already well explored [13,15,20,21]. The common disadvantages of using rodent AOM models comprise difficulties in small size tissue manipulation, in generating a multifactorial background of pathogenesis, and in some cases the development of sepsis (Table 1) [13,15,17,19,22,23,24,25].

AOM represents one of the most often cited reasons for the antibiotic prescription in children under 3 years of age [33]. This condition can be caused by both bacterial (*Moraxella catarrhalis*, *Staphylococcus aureus*, *Streptococcus pneumoniae* and *Haemophilus influenzae*) and viral pathogens (such as rhinovirus, respiratory syncytial virus, adenovirus, and influenza virus) [33,34,35]. A rigorous evaluation of the studies to verify whether antibiotic treatment impacts AOM outcomes is difficult to interpret, given the great rate of spontaneous recovery in children with this condition [36]. Thus, appropriate animal models that mimic the human infection have been designed to study this disease and to explore the efficacy of some antibiotic agents (Table 2).

A careful assessment of these studies in which these drugs were evaluated together with detailed analysis of pharmacodynamics and pharmacokinetic aspects of each of them could differentiate the various compounds and recognize those with best tolerability, cost/efficacy ratio, and safety.

## 3. Treatment Options, Antibiotic Resistance, and New Antibiotics/Antibiotics’ Schemes in AOM in Children

### 3.1. Classic Therapeutic Options in AOM in Children

The preferred therapeutic agents remain amoxicillin and the amoxicillin-clavulanate combination for AOM caused by *S. pneumoniae*, whereas oral cephalosporin’s (cefixime) is preferred in AOM due to *M. catarrhalis*, and *H. influenzae* [11]. The efficacy of amoxicillin-clavulanate versus azithromycin in 180 children with OMA aged 6 months to 12 years has been prospectively studied by Guven et al. [11]. The 10-day amoxicillin-clavulanate therapy (45 mg amoxicillin/kg) regimen was as effective as the azithromycin 3-day therapy (10 mk/kg/day, once). Both were well tolerated and had an equal incidence of side effects [11]. Kono et al. [12] have compared two “classic” antibiotic schemes in a sample of 146 pediatric patients with AOM (age 1 month to 5 years old) from eight private Japanese clinics: amoxicillin (70 mg/kg) for five days and amoxicillin in the same dose + clarithromycin (15 mg/kg) only for the first 3 days. *M. catarrhalis*, *S. pneumoniae* and *H. influenzae* were identified in the secretions from the middle ear. The curing of AOM was confirmed 14 days after the start of treatment for both groups. Therapeutic failure was recorded in 25 children, 16 of 58 being under 2 years of age. In the group treated with amoxicillin + clarithromycin, a smaller number of therapeutic failures were registered. Despite the proven efficacy of the conventional treatment, antibiotic resistance is an increasing phenomenon nowadays. A meta-analysis of the studies on the antimicrobial resistance in AOM in children performed during 1980–2017 [39], detected resistance of Gram-positive bacteria (*S. pneumoniae*) to penicillin but not to amoxicillin, the recommendation being to continue using this antibiotic as a first choice in treating children with otitis media [39,40]. The bacteriology of AOM was dominated by *S. pneumoniae* and *H. influenzae*, both of which were insensitive to first-line therapy. *M. catarrhalis* has demonstrated beta-lactamase production and amoxicillin resistance. In cases of penicillin allergy, erythromycin was used, for which *S. pneumoniae* showed high resistance. However, all three pathogens were susceptible to the amoxicillin-clavulanate combination, a combination that is normally recommended as second-line therapy [39]. Brook et al. [41] have reported beta-lactamase activity in 79% of the ear samples from children with otitis media. This might be explained by the fact that 70% of the patients had been treated with beta-lactam antibiotics [42].

### 3.2. Antibiotic Resistance

A ten-year retrospective analysis revealed that the rate of antibiotic-resistant ear infections has doubled over the last decade. Ampicillin had the highest overall resistance rate (88.5%), followed by ceftriaxone (84.5%), amoxicillin (81.9%), and tetracycline (74.5%) [43]. Although important advances have been made in the development of pneumococcal conjugate vaccines, multidrug resistance was found in 84% of the *S. pneumoniae* strains isolated in 62 children, aged between 1 and 16 years, during the episodes of AOM (including recurrent/treatment failure AOM and post-treatment visits) [44]. Bacterial resistance to antibiotics occurs frequently and is the ability of pathogens (bacteria, parasites, viruses, etc.) to develop in the presence of active substances that would normally destroy or inactivate them. Antibiotics identify bacteria and then exert their bactericidal or bacteriostatic effect [4,10]. The phenomenon of bacterial resistance occurs most often in the case of long-term, repetitive therapies, otitis media being the condition that often requires such therapy. Beta-lactamases are enzymes produced by many bacteria resistant to beta-lactam antibiotics (penicillins, cephalosporins, cefamycins, and carbapenems), when these antibiotics are present in the environment. Penicillinase is a specific type of beta-lactamase [10] released by Gram-negative anaerobic bacteria in the infected area.

**Figure 1:**
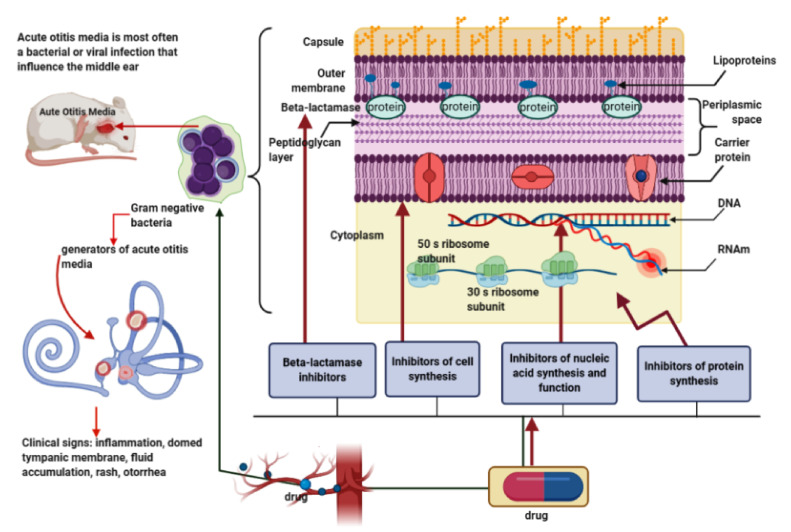
In the determination of otitis media, pathogens such as Gram-positive and -negative bacteria are causative agents of the acute form; the Gram-negative bacterium is represented in the image because, from a structural and chemical point of view, it is more complex compared with the Gram-positive one. The cell wall contains two outer layers of the cytoplasmic membrane and above the peptidoglycan layer is an outer membrane present only in Gram-negative bacteria. Specific for these bacteria are the “porins” (transmembrane proteins) that allow the passage of antibiotic molecules. Clinically, acute otitis media (AOM) is characterized by inflammation, bulging of the tympanic membrane, accumulation of purulent secretion, otorrhea, and discomfort. The effectiveness of antibiotics in this condition has been proven in both preclinical and clinical studies through various mechanisms of action that affect the middle ear, such as inhibition of cell synthesis, nucleic acids and disruption of their function, inhibition of protein synthesis, and enzymes such as beta-lactamases.

In order to understand the mechanism of bacterial resistance to beta-lactam antibiotics, we will shortly discuss the main mechanisms of action of penicillin-type antibiotics. These are antibiotics with a beta-lactam structure that selectively interfere with the synthesis of the bacterial cell wall, a wall made of a polymer called peptidoglycan. More specifically, it acts in the last stage of the synthesis of the bacterial cell wall, as it is a bactericidal process and consists in the irreversible binding of the beta-lactam ring in the antibiotic structure to the penicillin binding proteins (PBPs) located in the membrane, which serve as specific receptors for beta-lactams. All beta-lactam antibiotics, which inhibit glutamine synthetase, bind to PBPs and have been shown to catalyze a number of reactions involved in the process of synthesizing cross-linked peptidoglycan necessary to maintain the integrity of the bacterial cell. This is an irreversible reaction and inactivates the enzyme. These proteins (transpeptidases) are bacterial enzymes that bind penicillin, and their exposure to antibiotics prevents the synthesis of the cell wall and causes structural changes or even cell lysis. Penicillins inhibit this transpeptidase-catalyzed reaction, thus blocking cell wall synthesis and the accumulation of “Park nucleotide” or uridine diphosphate (UDP) -acetylmuramyl-pentapeptide. Transpeptidases or penicillin-binding proteins, in its presence, form the penicilloyl-enzyme complex with impaired deacetylation reaction catalyzed by transpeptidase with lysis and cell death, hence the quality of bactericidal agents [45]. Another mechanism for the establishment of antimicrobial resistance consists in the alteration of membrane receptors by the modification of the affinity of the receptor for bacteria, amplifying bacterial activity and canceling the activity of the drug [46,47]. Presented in Figure 1 is a schematic representation of the passage of antibiotic molecules in a Gram-negative bacterium.

### 3.3. New Antibiotics/Antibiotic Schemes in the Treatment of AOM in Children

The most effective therapies for beta-lactamase producing pathogens include clindamycin, chloramphenicol and metronidazole in combination with a macrolide, or the combinations of amoxicillin/clavulanate [41] or piperacillin/tazobactam [47]. Clavulanate is a beta-lactamase inhibitor that competes with the antibiotic to bind to the beta-lactamase, prolonging the life of beta-lactamase sensitive antibiotics that could be inactivated by bacteria if beta-lactamase inhibitors were not associated [42,48]. Carbapenem therapy (meropenem, imipenem) can be effective in both aerobic and anaerobic pathogens [49].

Suzuki et al. [4] evaluated six otorhinolaryngology infectious diseases in both adults and children, including chronic OM and AOM, and the antimicrobial susceptibility patterns of the isolated pathogens in 36 hospitals in Japan. Children under 6 years of age with AOM were subjected to antibiotic susceptibility tests for 40 antimicrobial agents from different classes, such as penicillins, cephemes, carbapenems, macrolides, tetracycline, new quinolones (Tosufloxacin and Levofloxacin), glycopeptides, oxazolidinones, aminoglycosides, lipopeptides, lincomycins, imidazoles, and fosfomycin. Among the 149 cases of recurrent AOM in children, *H. influenzae* was the most prevalent bacteria, followed by *S. pneumoniae* and *M. catarrhalis*. The antimicrobial susceptibility testing for the major bacterial strains revealed bacterial resistance for *S. pneumoniae* and *H. influenzae*, especially in patients with AOM, with a frequency that was not influenced by gender, age, geographical area, severity of the disease, hospital institution, and previous medication. The authors do not clearly set up in the end a first-line antimicrobial treatment, but they recommend amoxicillin or amoxicillin-clavulanate (in higher than usual dosage: 60–90 mg/kg of amoxicillin), cefditoren, or cefditoren-pivoxil for moderate cases only. Mild cases should receive only symptomatic treatment.

For resistant *S. pneumoniae*, new compounds such as Tebipenem pivoxil as the first carbapenem with oral administration available for children could be considered [4,50,51].

The efficacy of tebipenem-pivoxil in AOM in children has been demonstrated by Sugita et al. [52] in a clinical trial evaluating the pharmacokinetic–pharmacodynamic parameters at the site of action: the maximum concentration in the ear effusion exceeded the minimum inhibitory concentration for *S. pneumoniae* and *H. influenzae* isolates, thus indicating a favorable transfer of tebipenem to the overflow of the ear. The carbapenem efficiency has been proven and confirmed also by the low incidence of relapses and the high rate of the number of cases cured [52].

The therapy with non-beta-lactam compounds (macrolides and quinolones) has been widely used as an alternative to beta-lactams. The use of new quinolones such as tosufloxacin has increased due to macrolide resistance. Tosufloxacin has been used in children with high antimicrobial activity against *H. influenzae*. Garenoxacin and Moxifloxacin are recommended only in adults [4]. However, the efficacy of tosufloxacinon isolates taken from pediatric patients is still discussed. In the study of Tanaka et al. [53] *H. influenzae* isolates have been proved resistant and can only survive at the maximum therapeutic concentration of tosufloxacin [53]. Several studies reported a decreased sensitivity to clarithromycin and revealed numerous quinolone-resistant strains [54,55,56].

Cefditoren pivoxil, a third-generation cephalosporin, is administered as an oral prodrug, absorbed and metabolized by hydrolysis reaction in the active compound cefditoren in the intestine, distributed in the blood until the site of action, and then eliminated by the kidneys [57]. It has a broad spectrum of activity, on Gram-positive and Gram-negative bacteria [58], inhibiting the cell wall synthesis by binding the PBP, followed by loss of cell wall integrity and cell death [57]. The studies on pharmacokinetic modeling and pharmacodynamics profile of cefditoren in the presence of human albumin, serum, plasma, and epithelial lining fluid [59] have demonstrated that the bactericidal activity of this compound could be influenced by the ratio between the total serum concentration of the bound form and the free form [60]. Several studies have shown that cefditoren had a very strong effect against *H. influenzae*, regardless of β-lactamase production [57,61].

## 4. Guidelines for Antibiotic Treatment in AOM in Children

In order to reduce the potential complications (e.g., mastoiditis, meningitis, and hearing loss) and the economic burden associated with AOM, various professional guidelines and consensus papers have been created. In the United States of America, Sweden, United Kingdom, France, Spain, and Italy, the introduction of guidelines was associated with a reduction of up to 12% of unnecessary prescriptions and an increase of up to 58% in the accuracy of the type and dosage of the prescribed antibiotic [62]. According to a recent Italian study, the implementation of guidelines in a pediatric emergency department led to a reduction from 53.2% to 32.4% in the use of broad-spectrum antibiotics [63]. We proposed to identify and analyze the available guidelines on AOM to detect common aspects, as well as differences, with a focus on antibiotic prescription, recommendations regarding watchful waiting approach, and follow-up strategies.

A systematic review of the literature for guidelines published between January 1989 (first AOM guideline, Netherlands) and September 2020 was conducted. We performed a systematic search on PubMed and EMBASE databases using keywords: “acute otitis media” AND “children” OR “pediatric” AND (“guideline” OR “consensus”). National or international clinical practice guidelines and guidance documents with recommendations for the management of AOM in children were eligible for inclusion. A total of 263 papers have been initially found. The following items were excluded: withdrawn or superseded guidelines, clinical trials or systematic reviews not part of clinical practice guidelines, and types of publications other than guidelines or consensuses (e.g., case reports or case series). Websites of national pediatric associations and bibliographies of all included guidelines were examined in order to identify further relevant resources.

A total of 20 AOM clinical practice guidelines were included in this review. The following guidelines were analyzed and compared regarding antibiotic treatment options: World Health Organization (WHO), Finland, U.S., Australia, Czech Republic, France, Italy, Spain, Denmark, Poland, Portugal, U.K., Belgium, Germany, Ireland, Luxembourg, Netherlands, Norway, Sweden, and Switzerland. All guidelines emphasize the need for accurate diagnosis, which is the main condition in order to establish the adequate treatment.

Two types of approaches towards antibiotic administration have been identified: a watchful waiting approach and immediate antibiotic prescription. WHO guideline recommends antibiotics to all children with confirmed AOM. Immediate antibiotics for any AOM can be considered according to Finland, U.S. and Czech Republic guidelines, whereas the rest of the guidelines mentioned above (16/20) encourage the watchful waiting approach. The indications for immediate antibiotic treatment are also based on the presence of severe symptoms (fever, otalgia, pain, vomiting, diarrhea, tympanic membrane perforation, or otorrhea) and the recurrence of AOM episodes (Table 3). The advantages of the watchful waiting approach are both economic, in terms of cost savings, and also microbiological by preventing the emergence of antibiotic resistance [64]. The decision of whether to immediately recommend antibiotics or to allow the patient an observation period of 48 to 72 h in order to obtain a spontaneous clearing of the OM infections is based on an accurate diagnosis. As there is no available gold standard diagnostic test, the guidelines aim to provide greater precision in the diagnosis and treatment of AOM.

Seventy-five percent (15/20) of the guidelines include young age as a criterion for immediate antimicrobial treatment; 57% (8/14) of them recommend antibiotic therapy for children younger than 24 months old. Only 20% (4/20) of the guidelines recommend antibiotics as a first-choice treatment in children with unilateral AOM, whereas 75% (14/20) of guidelines support immediate antibiotic treatment in cases of bilateral AOM in children aged less than 2 years. The decision to prescribe antimicrobial treatment as a first-line choice is based on the presence of severe symptoms (fever, otalgia, pain, vomiting, and diarrhea) in 85% (17/20) of the guidelines included in this review.

Recommendations regarding the management of recurrent AOM (three or more episodes of AOM in the previous 6 months or four or more episodes in the last 12 months) are included in 40% (8/20) of the guidelines included in this review. Long-term antibiotics are not recommended routinely. The Australian guidelines encourage treatment with long-term antibiotics (e.g., amoxicillin 25–50 mg/kg 1–2 times daily) for 3–6 months in children <2 years of age who are at risk of developing chronic suppurative otitis media. Antibiotic stewardship is promoted by providing clear diagnostic criteria (18/20 guidelines, except Ireland and Switzerland) and by clearly specifying the moment when to initiate antimicrobial treatment (20/20). Almost all the guidelines (19/20, except Ireland’s guideline) clearly specify the duration of antibiotic regimens. Amoxicillin is universally accepted as first-line antibiotic therapy in all included guidelines (Table 4). There is a wide variety of suggested antibiotics as second- and third-line treatments, as well as for the duration and dosage of therapy. The choice of amoxicillin is based on consideration of etiological spectrum of AOM and data on antibiotic resistance. The benefits of antibiotic treatment for AOM consist of the management of pain and the reduction in the risk of serious, acute complications as well as the risk of the long-term sequelae, including chronic suppurate otitis media and mastoiditis.

After the introduction of national AOM clinical practice guidelines the antibiotic prescription rates decreased by 12% with an increase of up to 58% for the recommended first choice antibiotic [62]. Since the introduction of the first AOM management guideline in 1989 [85], various professional guidelines for AOM diagnosis and treatment have been published. The first review that attempted to compare the current guidelines in AOM management revealed that most guidelines recommend amoxicillin as first-line antibiotic, whereas options for second-line and third-line therapies varied. The authors analyzed the guidelines from selected developed and developing countries, explaining that the differences occur due to differences in local epidemiology, healthcare policy, accessibility to health facilities, and health expenditure [86]. The most recent and compressive review on clinical practice guidelines for AOM in children reveal that there are major similarities in AOM management recommendations in the included papers. In addition to the approach to antibiotic administration (immediate antibiotic treatment, options for first- or second-line antibiotics), the review also compares the methodological quality of the included guidelines, the diagnosis criteria, and whether or not these papers include country-specific resistance patterns for antibiotic administrations [87]. To discuss each of these aspects was beyond the purpose of our paper. However, we noticed some similarities between our results and the conclusions of this article regarding the common aspects discussed in both papers. Oral amoxicillin was the first-line treatment option. Almost the same criteria for immediate antibiotic recommendations were analyzed: age, unilateral AOM, bilateral AOM in children <2 years old, tympanic membrane (TM) perforation/otorrhea, and recurrent AOM. In addition to these criteria, Suzuki et al. included the comorbidities and the parental input. In contrast to this review, we attempted to offer detailed comparison of antimicrobial treatment recommendations, including not only the first-line options but also the suggested alternatives (second-line/treatment failure, third-line/allergy to first-line), in order to provide an orientation tool for clinicians.

## 5. Concluding Remarks

*S. pneumoniae* and *H. influenzae* remain the most important pathogens in the determination of AOM, being the bacteria against which antimicrobial treatment should be targeted. The rat, mouse, gerbil, guinea pig, and chinchilla are the preferred animals for experimental AOM models with individual advantages and disadvantages. Antibiotic resistance is rising in all parts of the world due to the misuse and overuse of the antimicrobial treatment. In response of this worrying phenomenon, new antibiotics have been proven efficient in AOM in children: Tebipenem-pivoxil, Tosufloxacin, and Cefditoren pivoxil. Overall evidence suggests that guideline adherence is effective in reducing antibiotic prescriptions. A total of 20 AOM clinical practice guidelines were included in this review: World Health Organization (WHO), Finland, U.S., Australia, Czech Republic, France, Italy, Spain, Denmark, Poland, Portugal, U.K., Belgium, Germany, Ireland, Luxembourg, Netherlands, Norway, Sweden, and Switzerland. The current guidelines on AOM management include two types of approaches towards antibiotic administration: watchful waiting approach and immediate antibiotic prescription. Amoxicillin is proposed as first-line antimicrobial therapy in all included guidelines, whereas cephalosporin, amoxicillin-clavulanic acid, or Trimethoprim-sulfamethoxazole are among the second-line antibiotics. Recommended treatment duration varied from 5 to 10 days. A higher dosage than usual is recommended for amoxicillin and amoxicillin-clavulanic acid in order to overcome resistant strains. Sixteen of the guidelines recommend immediate antibiotic treatment in case of severe symptoms (fever, otalgia, pain, vomiting, and diarrhea). Fifteen of the 20 included guidelines include age as a criterion for immediate antimicrobial treatment, eight of them recommending immediate antibiotic therapy for children younger than 24 months old. Judicious use of antibiotics is a priority, as antimicrobial resistance represents a serious threat to global public health and the adherence to guideline may reduce the treatment failures, which are usually related to antibiotic resistant strains.

## Figures and Tables

**Table 1:** The most common advantages and disadvantages of rodent species used in acute otitis media models.

**MICE** [17,19,20,26,27]
Advantage: animal small size, low husbandry cost, easy handling; availability of transgenic and knockout strains; a well-defined immune system, genetic tractability and a well-described microbiological status; availability of appropriate reagents. Disadvantage: small size of middle ear and thin tympanic membrane; anesthetic drugs susceptibility; large and patent Eustachian tube.
**RATS** [15,22,23,28]
Advantage: the opening Eustachian tube pressure corresponds to that in humans; anatomy and histology of middle ear compatible with that of the human children; medium size tympanic bulla; not susceptible to sepsis; pharmacokinetic profile and gene. Disadvantage: capable of developing spontaneous acute otitis media.
**CHINCHILLA** [24,25]
Advantage: large bulla that allows for an easy inoculation of pathogens; tympanic membrane almost the same dimension as in humans; rarely develops natural acute otitis media; anatomy and histology of middle ear compatible with that of the human children; susceptible to humans pathogens of the middle ear. Disadvantage: prone to developing general sepsis accompanied by a high mortality rate.
**GERBIL** [29,30,31]
Advantage: a relatively large middle ear; a small incidence of spontaneous acute otitis media; susceptible to humans pathogens of the middle ear; animal small size, low husbandry cost, easy handling. Disadvantage: tight external auditory canal.
**GUINEA PIG** [13,32]
Advantage: easy middle ear inoculation of pathogens. Disadvantage: narrow external auditory canal and reduced middle ear volume; differences in the anatomy and histology of middle ear, pharmacokinetic parameters and immune status; difficulty in generating otitis media.

**Table 2:** Preclinical assessment of antibiotics in animal models of acute otitis media.

Types of Animals and Author	Causative Pathogen and Route of Administration	Antibiotic Therapy	Results
**RATS**			
Piltcher et al. [23]; Hebda et al. [28]; Genc et al. [25]; Ucar et al. [37]	transbullar inoculation of *Streptococcus pneumoniae,* type 6A; transbullar inoculation of *Streptococcus pneumoniae*, type 6A; middle ear inoculation of type 3 *Streptococcus pneumoniae;* transtympanic suspension of type 3 *Pneumococci*	intramuscular delivery of 100 mg/kg ampicillin twice daily for 5 days; treated twice daily from day 2 to day 7 with 100 mg/kg ampicillin in gavage delivery; a single daily dose of 100 mg/kg clarithromycin in gavage delivery for 5 weeks; intramuscular injection of 160.000 UI/kg/day penicillin G for 5 days	Experimental design induced reproducible pathologic signs similar to those for OME, and *Streptococcus pneumoniae* was not recovered on or after day 7 suggests that the therapy introduced efficiently sterilized the middle ear cleft; Both effusion cytokine levels (IL-1β, IL-6, IL-10, TNF-α and MIP-2) and mucosal cytokine transcripts (IL-1β, TNF-α, IL-6, IL-10, IFN-γ, TGF-β1, MCP1, IL-8) were much less after antibiotic therapy; The drug decreased the tympanosclerosis severity determined by acute otitis media and myringotomies; Favorable effects on the mucosal changes of the middle ear
**CHINCHILLA**			
Post et al. [21]; Yang et al. [24]; Yang et al. [38]	transbullar inoculation of non-typable *Hemophilus influenzae;* transnasal inoculation of *Streptococcus pneumoniae;* transbullar inoculation of nontypable *Haemophilus influenzae*	intramuscular ampicillin therapy twice daily for 4 days, 50 mg/kg/per dose; topical delivery of 1 to 4% ciprofloxacin –3CPE-(P407-PBP) hydrogel formulation transtympanic delivery of 2 mg of ciprofloxacin hydrogel formulation	In the samples harvested at 120 h after ampicillin treatment, no obvious bacterial biofilm was observed, even if individual bacteria were again easy to identify. A single transtympanic application of 4% ciprofloxacin-3CPE-[P407-PBP] hydrogel was able to cure *Streptococcus pneumoniae.* The hydrogel system completely eliminated otitis media in all animals tested, while only 62.5% of chinchillas who received 1% ciprofloxacin alone monotherapy eradicated the infection by day 7. The drug delivery system was biocompatible in the ear and ciprofloxacin was undetectable in the blood, suggesting an adequate local drug distribution and activity
**GUINEA PIGS**			
Bruk et al. [13]; Wang et al. [32]	transbullar injection of non-typeable *Haemophilus influenza;* middle ear; inoculation of *Streptococcus pneumoniae*	a single topical delivery of ciprofloxacin (10 mg) and ceftriaxone (30 mg)-loaded polymer microspheres and thermoresponsive gel; intratympanic injection of various doses of ciprofloxacin hydrogel 0.06% to 12% (containing ciprofloxacin in 16% poloxamer 407	Ciprofloxacin microspheres/gel therapy resulted in a significant reduced bacterial count on days 7 and 14 post-inoculation, with complete clearance detected on day 14. Ceftriaxone microspheres/gel treatment resulted in an important decreased infection after 7 days of therapy, with a recurrence of infection by day 14. A single intratympanic delivery of ciprofloxacin hydrogel offers an equal therapy effect against OM as a twice daily multiday regimen of Ciprodex (0.3% ciprofloxacin and 0.1% dexamethasone suspension) or Cetraxal (0.2% ciprofloxacin solution) topical drops

OME, otitis media with effusion; IL-1β, interleukin-1β; IL-6, interleukin-6; IL-8, interleukin-8; IL-10, interleukin-10; TNF-α, tumor necrosis factor- alpha; TGF-β1, transforming growth factor-β1; IFN-γ, interferon-γ; MCP1, monocyte chemoattractant protein-1; MIP-2 protein, macrophage inflammatory protein-2; 4% ciprofloxacin-3CPE-(P407-PBP), 4% (*w*/*v*) ciprofloxacin, 2% (*w*/*v*) limonene, 1% (*w*/*v*) sodium dodecyl sulfate, 0.5% (*w*/*v*) bupivacaine hydrochloride, and 12% (*w*/*v*) poloxamer 407-polybutylphosphoester; OM, otitis media; transbullar approach, a direct delivery bacterial inoculation method where the middle ear can be inoculated by a fine needle through the bony bulla wall.

**Table 3:** Indications for immediate antibiotic treatment.

Country Guideline	Age (Months)	Unilateral AOM	Bilateral AOM in Children	Severe Symptoms	TM Perforation/Otorrhoea	Recurrent AOM
WHO [65]	All age groups	Yes	Yes *	Yes	Yes	-
USA [66]	-	Yes	Yes *	Yes	-	Yes
Australia [67]	<24	-	Yes *	Yes	Yes	Yes
UK [68]	All age groups #	-	Yes *	Yes	Yes	-
Italy [69]	-	Yes	Yes *	Yes	Yes	-
Spain [70]	<24	-	Yes *	Yes	Yes	Yes
Denmark [71]	<6	-	Yes *	Yes	-	Yes
France [72]	<24	-	-	Yes	-	-
Portugal [73]	<6	-	Yes *	Yes	Yes	Yes
Norway [74]	<12	-	Yes *	-	Yes	-
Germany [75]	<24	-	Yes *	Yes	Yes	Yes
Ireland [76]	-	-	-	-	Yes	-
Luxembourg [77]	<24	-	Yes **	Yes	-	-
Netherlands [78]	<6	-	Yes *	Yes	Yes	-
Finland [79]	<24	-	Yes ***	-	Yes	-
Belgium [80]	<6	-	Yes ***	Yes	Yes	-
Poland [81]	<6	Yes	Yes *	Yes	Yes	Yes
Sweden [82]	<12	-	Yes ***	Yes	Yes	-
Switzerland [83]	<24	-	Yes *	Yes	Yes	Yes
Czech Republic [84]	-	-	-	Yes	Yes	-

WHO, World Health Organization; #, with otorrhea; *, in children aged < 24 months of age; **, after consultation with parents; ***, bilateral AOM at any age; -, no recommendations in the guideline for the selected criteria.

**Table 4:** Antimicrobial treatment recommendations.

Country Guideline	First Line Antibiotic and Duration	Second line/Treatment Failure	Third Line/Allergy to First Line
WHO	PO amoxicillin, 80 mg/kg/day in 2 divided doses for 7–10 days	Repeat antibiotics for another 5 days	Not specified
USA	PO amoxicillin, 80 mg/kg/day in 2 divided doses; <2 years of age: 10 days; 2–5 years of age: 7 days; >6 years of age: 5–7 days	Treatment failure: Amoxicillin-clavulanate 90 mg/kg/day in 2 divided doses	Penicillin allergy: Cefdinir 14mg/kg per day per day/in 2 divided doses, cefuroxime 30 mg/kg/day in 2 divided doses, cefpodoxime 10 mg/kg/day in 2 divided doses, ceftriaxone 50 mg IM or IV/day for 1–3 days
Australia	PO amoxicillin 50 mg/kg in 2 divided doses in AOM without middle ear discharge for 7 days	High doses (e.g., amoxicillin 90 mg/kg) if failure to respond to standard	Not specified
	PO amoxicillin 50–90 mg/kg in 2 divided doses or in 3 divided doses in AOM with perforation for 14 days		
UK	PO amoxicillin, 1–11 months of age: 125 mg in 3 divided doses 1–4 years: 250 mg in 3 divided doses 5–7 days	Treatment failure: Amoxicillin-clavulanate	Penicillin allergy: Clarithromycin or erythromycin; dosage dependent on age
Italy	Mild symptoms and no otorrhea nor risk factors: PO Amoxicillin, 10 days < 2 years of age; 50 mg/kg/day in 2 divided doses or in 3 divided doses, 5 days <2 years of age; Severe symptoms, otorrhea, or risk factors for bacterial resistance Amoxicillin-clavulanate 80–90 mg/kg/day in 2 divided doses or in 3 divided doses	Treatment failure: If treated with amoxicillin or cefaclor: amoxicillin + clavulanate or cefpodoxime proxetil or cefuroxime axetil. If treated with a broad-spectrum antibiotic: IM or IV ceftriaxone 50 mg/kg per day	Penicillin allergy: macrolide
Spain	PO Amoxicillin 80–90 mg/kg/day in 3 divided doses for 5 days	Treatment failure: Amoxicillin-clavulanate 80–90 mg/kg/day in 3 divided doses for 7–10 days, IM/IV Ceftriaxone 50 mg/kg/day in 3 divided doses	Penicillin allergy: Cefuroxime axetil 30 mg/kg/day in 2 divided doses
Denmark	PO Penicillin V, 60 mg/kg/day in 3 divided doses for 7 days	Treatment failure: <2 years of age: Amoxicillin-clavulanate 10/2.5 mg/kg/dose in 3 divided doses for 7 days; 2–12 years of age: amoxicillin-clavulanic acid 1-/2.5 mg/kg/dose 8 h for 7 days	Penicillin allergy: clarithromycin 7.5 mg/kg/dose x 2 for 7 days
France	PO amoxicillin 80–90 mg/kg/day in 2–3 divided doses; >2 years of age: 5 days, <2 years of age: 8–10 days	Treatment failure: PO Amoxicillin/clavulanate 80 mg/kg/day and PO amoxicillin 70 mg/kg/day, IM/IV ceftriaxone 50 mg/kg daily for 3 days	Allergy to beta lactams: erythromycin-sulfafurazole or cotrimoxazole. Allergy to penicillin’s without allergy to; cephalosporin’s: cefpodoxime
Portugal	Amoxicillin 80–90 mg/day in 2 divided doses; 5 days	Treatment failure: PO/IV Amoxicillin and clavulanate 80–90 mg/kg/day in 2 divided doses, PO Cefuroxime-axetil 30 mg/kg/day in 2 divided doses, IV 80–100 mg/kg/day in 3 divided doses 7 days if <2 years, IM/IV Ceftriaxone 50 mg/kg/day once daily	Penicillin allergy: Clarithromycin 50 mg/kg/day in 2 divided doses or Erythromycin 50 mg/kg/day in 3–4 divided doses per day or Azithromycin 10 mg/kg/day once a day
Norway	PO phenoxymethylpenicillin 24–60 mg/kg/day in 3–4 divided doses per day for 5 days	Treatment failure: trimethoprim sulfamethoxazole	Allergy: Erythromycin or Clarithromycin (children over 6 months)
Germany	PO Amoxicillin 50 mg/kg/day in 2–3 divided doses. If from country with high rates of penicillin resistance: PO amoxicillin 80–90 mg/kg/day for 7 days	Treatment failure: PO amoxicillin 80–90 mg/kg/day; Second choice: PO cephalosporin including cefuroxime axetil (20–30 mg/kg/day for 5 days)	Allergy to penicillin’s/ cephalosporin’s: erythromycin 7 days
Ireland	Amoxicillin (no specifications regarding duration, route or frequency)	Not specified	Not specified
Luxembourg	Amoxicillin 80–90 mg/kg/day in 3 divided doses; <6 years 10 days treatment >6 years 5–7 days treatment	Treatment failure: Amoxicillin/clavulanate; Otherwise cefuroxime axetil, ceftriaxone 50 mg/kg/day for 3 days, azithromycin, Clarithromycin, clindamycin	If vomiting: Ceftriaxone 50 mg/kg once daily for 3 days. Penicillin allergy: Cefuroxime 30 mg/kg in two divided doses
Netherlands	Amoxicillin 40 mg/kg/day in 3 divided doses, for 7 days	Second line and also treatment failure: Amoxicillin-clavulanic acid 40/10 mg/kg/day in 3 divided doses for 7 days	Penicillin allergy: cotrimoxazole 36 mg/kg/day in two divided doses; 5–7 days
Finland	PO Amoxicillin 40 mg/kg/day 8–12 h/PO amoxicillin-clavulanate 40/5.7 mg/kg/day in 2–3 divided doses; 5–7 days	If vomiting: IM Ceftriaxone (one dose)	Penicillin allergy: cefaclor, cefuroximexetil, sulfamethoxazole-trimethoprim, azithromycin or clarithromycin
Belgium	PO amoxicillin 75–100 mg/kg/day in 3 divided doses for 7 days	Treatment failure: cefuroxime axetil 30–50 mg/kg in 3 doses amoxicillin-clavulanate 50/37.5 mg/kg in 3 doses	In case of Allergy to cephalosporin’s: co-trimoxazole (Trimethoprim 8 mg/kg/day and Sulfamethoxazole 40 mg/kg/day) in 3 divided doses or Levofloxacin 10 mg/kg/day in 2 divided doses

WHO, World Health Organization; PO, per os; IM, intramuscularly; IV, intravenous.

## Data Availability

No new data were created or analyzed in this study. Data sharing is not applicable to this article.

## Abbreviations

AOM	acute otitis media
BLPB	bacterial beta-lactamase enzyme
IFN-γ	interferon-γ
IL-10	interleukin-10
IL-1β	interleukin-1β
IL-6	interleukin-6
IL-8	interleukin-8
IM	intramuscularly
IV	intravenous
MCP1	monocyte chemoattractant protein-1
MIC	the minimum inhibitory concentration
MIP-2 protein	macrophage inflammatory protein-2
OME; OM	otitis media with effusion; otitis media
PBP	penicillin binding proteins
PO	per os
TGF-β1	transforming growth factor-β1
TM	trauma severity score
TNF-α	tumor necrosis factor- alpha
UDP	uridine diphosphate
WHO	World Health Organization
